# Cornea As a Model for Testing CTGF-Based Antiscarring Drugs

**DOI:** 10.4137/BTRI.S19954

**Published:** 2016-06-20

**Authors:** Sriniwas Sriram, Jennifer A. Tran, James D. Zieske

**Affiliations:** 1 Schepens Eye Research Institute, Boston, MA, USA; 2 Department of Ophthalmology, Massachusetts Eye and Ear, Harvard Medical School, Boston, MA, USA

**Keywords:** CTGF, corneal scar, skin scarring, scarring treatments

## Abstract

Scarring remains a serious complication of the wound healing process that can lead to the formation of excessive fibrous connective tissue in an organ or tissue leading to pain and loss of function. This process is mainly regulated by Transforming growth factor β1 (TGF-β1), which binds to receptors and induces its downstream mediator, Connective tissue growth factor (CTGF). The number of drugs targeting CTGF for treating scars has been on the rise in the past few years. The purpose of this article is to suggest the possibility of using cornea as a model for testing anti-CTGF therapies for scarring.

Wound healing is a complex and dynamic process that replaces devitalized and missing cellular structures or tissue layers after an injury and involves multiple interactions between various growth factors and their signaling pathways. It can be generally divided into three phases: inflammatory, proliferative, and maturation.^1^ The culmination of all these processes results in the replacement of normal tissue with a fibroblast-mediated regenerated tissue. One undesirable side effect of wound healing is scarring, which is the formation of excess fibrous connective tissue in an organ or tissue. This excess tissue may physiologically act to obliterate the architecture of the underlying organ or tissue leading to pain and loss of function. Although the skin is the most well-known example of scarring, all of the body's tissues are capable of scarring.

Developing antiscarring treatments has not been forthcoming because the spectrum of wound healing ranges from complete regeneration of tissue to the formation of hypertrophic and keloid scars. Any interruption or failure to heal completely may lead to the formation of nonhealing chronic wounds. The difficulty in developing an antiscarring treatment lies in maintaining this delicate balance between healing and regeneration. Although it may be possible to eliminate scarring by blocking a major signaling cascade involved in wound healing, it would also adversely affect the ability of the wound to heal completely. Finding this balance is the key to therapeutically modulate the healing response and reduce the severity of subsequent scarring.

## Potential Antiscarring Treatments that Target Connective Tissue Growth Factor are on the Rise

Treatments for the reduction of scarring represent a clear unmet medical need. Currently, there are no registered pharmaceuticals for the prophylactic improvement of scarring, and no single therapy is universally accepted as the standard of care. The US potential market for skin scarring treatments is estimated to be up to $4 billion with approximately 42 million surgical procedures conducted annually.^2^ Many companies have joined the race to develop antiscarring/antifibrotic therapies that can prevent unsightly and debilitating dermal scars as well as to treat other medical conditions where excess fibrosis may occur. In 2011, Pfizer, a pharmaceutical giant, acquired Excaliard Pharmaceuticals Inc., a company founded on the basis of developing treatments against skin fibrosis.^3^ Another company, Fibro-Gen, is enrolling patients to run clinical trials of their human monoclonal antibody in individuals with idiopathic pulmonary fibrosis.^4^ In addition, RXi Pharmaceuticals has shown promising results with their RNA interference-based dermal antiscarring drug candidate.^5^ These prophylactic treatments involve agents that are administered locally at the time of surgery or injury and may lead to long-term improvements in scarring. All three companies target the reduction of a growth factor called Connective tissue growth factor (CTGF). The overexpression of CTGF is correlated with severe fibrotic disorders in different parts of the body, including fibrosis in skin, kidney, liver, lung, eye, and vasculature.^6^ It acts as a downstream mediator of Transforming growth factor beta-1 (TGFβ-1), leading to the transformation of fibroblasts to scar-forming myofibroblasts. The idea that different companies are targeting CTGF to counter scarring raises hopes that, eventually, a single therapy can be developed to counter fibrosis in different parts of the body.

## Cornea as a Model for Scarring

The first step to understand this balance may be to study a simplified model of wound healing. In many ways, the cornea can be considered as such. Just as scar tissue can form on a person's skin, it can also form on the cornea, most commonly after an infection, trauma, or due to severe chemical burns. The molecular pathways governing wound healing is comparable to that of skin and involves major players such as TGFβ-1 and CTGF. There are also other advantages to testing antiscarring drugs in the cornea when compared with the other parts of the body. Since the cornea is normally avascular, the complexities of the wound are absent. Additionally, the structure and immune-privileged nature of the cornea makes it easier to introduce foreign substances without eliciting an immune response.^7^ This would greatly simplify the drug delivery problems encountered in the other parts of the body like the kidney or lung. Finally, there are a number of established *in vitro*, *ex vivo*, and *in vivo* models already developed for the cornea.^8–10^

An effective antiscarring treatment would also provide immediate benefits to those suffering from visual impairments due to corneal fibrosis. There are an estimated 20 million refractive surgical procedures performed worldwide each year, mainly consisting of LASIK surgery for myopia. Although complications are low (in the 1%–2% range), there is a need for a nontoxic drug treatment to reduce scarring in those patients who develop corneal scars. The corneal scarring system can be used as an important aid for the screening and optimization of new antiscarring drugs, which could pave the way toward the development of an efficient antiscarring drug for the other parts of the body.
